# Content validation and use of mothers on respect index to determine levels of respectful maternity care among women facing disadvantage, birthing in the Top End of the Northern Territory: a cross-sectional study

**DOI:** 10.3389/fgwh.2025.1531904

**Published:** 2025-04-03

**Authors:** Emily Rebecca Bowden, Maree R. Toombs, Robyn L. Williams, Anne B. Chang, Deborah Richards, Meredith Porte, Stephanie Yerkovich, Gabrielle Britt McCallum

**Affiliations:** ^1^ Child and Maternal Health Division, Menzies School of Health Research, Charles Darwin University, Darwin, NT, Australia; ^2^ School of Population Health, University or New South Wales, Sydney, NSW, Australia; ^3^ Australian Centre for Health Services Innovation, Queensland University of Technology, Brisbane, QLD, Australia; ^4^ Department of Respiratory and Sleep Medicine, Queensland Children’s Hospital, Brisbane, QLD, Australia; ^5^ Department of Computing, Faculty of Science and Engineering, Macquarie University, Sydney, NSW, Australia

**Keywords:** respectful maternity care, migrant, refugee, First Nations, MORi, Northern Territory, systemic disadvantage

## Abstract

**Introduction:**

Although recognised as important, few validated tools are available to measure respectful maternity care. In Australia, First Nations, migrant and refugee women have fewer antenatal attendances and poorer outcomes compared to others, with disrespectful maternity care a known barrier to care-seeking. Our primary aim was to determine content validity of the Mothers on Respect index (MORi) for use with women facing disadvantage birthing in the Top End of the Northern Territory. Our secondary aim was to determine the extent of respectful maternity care amongst these women in our setting.

**Methods:**

Fifteen First Nations women participated in an iterative process, rating and commenting on the original MORi items using content-validation-index for items. 195 First Nations, migrant, refugee women subsequently completed the content-validated MORi, within 12-months postpartum.

**Results:**

Content validity was established for all items; The overall median MORi score was high at 78 [interquartile range (IQR) 72–83]. Migrant women had the highest median score of 80 (IQR 76–83), remote-living First Nations women had the lowest at 63.5 (IQR 55–76). There were no significant differences across antenatal attendance, educational attainment, or primary caregiver.

**Discussion:**

Overall, high levels of respectful maternity care were observed. First Nations women from remote communities, and refugee women within some domains, experienced lower levels of respect than others, perhaps resulting from ongoing systemic disadvantage. MORi content-validity was established for First Nations Australians, migrant and refugee women with disparity between cohorts observed. Continuity-of-carer, increased access to interpreters, and companion of choice may address some of these disparities.

## Introduction

The World Health Organization (WHO) defines respectful maternity care as “*care organized for and provided to all women in a manner that maintains their dignity, privacy, and confidentiality, ensures freedom from harm and mistreatment, and enables informed choice and continuous support during labour and childbirth*” (1). This definition takes a human rights approach, emphasizing the importance of treating women with respect, ensuring their autonomy and agency, and protecting their rights to privacy and confidentiality throughout their pregnancy journey.

Providing respectful maternity care is widely recognized as an important goal, particularly for women living with systemic disadvantage (2). While a number of tools have been developed for use in low-middle income countries to measure women's experience of respectful maternity care (3, 4), few have been developed and validated for use in high-income countries. However, one such tool, the Mothers on Respect index (MORi) (5), developed in Canada is gaining popularity, having recently been used successfully in Canada (5), Australia (6), and the Netherlands (7). It was designed to measure respectful care across the continuum of a woman's pregnancy, including birth and the postpartum period. The development process involved several rounds of testing and validation with women from diverse socio-cultural backgrounds.

An Australian study recently used the MORi on the East Coast and recommended its use to measure and improve respectful maternity care in Australia, and in other countries with similar maternity care settings (6). They content-validated the MORi with 10 women, followed by a larger cohort of 161 and reported high internal consistency, reliability and validity (4). However their cohort had a preponderance of Caucasian women (*n* = 141, 87.6%), and a small number of Aboriginal and Torres Strait Islander people, hereafter respectfully referred to as Australian First Nations women (*n* = 4, 2.5%). Participants in that study (6), while representative of the demographics of the region where it was conducted, inadequately represents the different geographical regions across Australia such as the Top End of the Northern Territory (NT). To facilitate the culturally secure inclusion of First Nations women, content validation of MORi is required.

It is well documented that Australian First Nations women are less likely to commence pregnancy care in the first trimester, attend the recommended number of antenatal visits (9), and are more likely to experience severe maternal morbidity or maternal mortality than non-First Nations women (10). A study conducted in South Australia found that First Nations women with more identified risk factors were less likely to perceive their care as having met their needs than women with fewer identified risk factors (13). While there is little data from the NT, other Australian jurisdictions have established that women from a migrant or refugee background are also less likely to be engaged in maternity services, resulting in poorer perinatal outcomes (11–13). Further, women from a migrant or refugee background report multiple barriers to service utilisation including lack of trust in health care providers, limited access to interpreters, and conflicts between cultural expectations and the Australian model of care (8). These are at odds with the WHO definition of disrespectful maternity care as described above (1).

While disrespectful maternity care is a recognised barrier to seeking care in pregnancy globally (14–16), little is known about levels of respectful maternity care received by First Nations women and women from a migrant or refugee background in the NT. Measuring respectful maternity care, therefore, is a critical area of research, particularly for Australian First Nations women, where historical and ongoing colonisation, discrimination, and trauma have led to significant mistrust of the healthcare system (17), or for women from a migrant or refugee background who also shoulder a disproportionate burden of systemic disadvantage (12, 13). With a large proportion of the NT population falling into one of these two cohorts (First Nations = 26.3%; born overseas = 23%) (15) and thus more likely to experience poorer pregnancy outcomes, it is important to determine the level of respectful maternity care in our setting to understand the needs and priorities of all pregnant and birthing women. The aim of this current study is thus to determine the extent to which women facing disadvantage, birthing in the Top End of the NT experience respectful maternity care by: (1) modifying and validating the content of the current MORi; and (2) administering the MORi to First Nations, migrant and refugee women.

### About the authors

The present study was conducted and authored by a diverse team of researchers with extensive experience working in various capacities across Australia. More details about the authors can be found in the Supplementary File.

## Materials and methods

### Ethics

This study adheres to the National Health and Medical Research Council (NHMRC) guidelines on the ethical conduct in research with Aboriginal and Torres Strait Islander Peoples and communities. Ethics approval was received from the Human Research Ethics Committee of NT Health and Menzies School of Health Research (HREC 2021-4205) on 21/12/2021. Prior to the commencement of the study, the methodology was reviewed and endorsed by the Menzies School of Health Research Australian First Nations Reference Group for Child and Maternal Health (hereafter referred to as “First Nations reference group”), with regular discussions throughout the study. The First Nations Reference Group is an overarching committee intended to provide high level strategic advice and advocacy on First Nations health research issues, advise on research priority setting in health research, maintain an overview of current research studies and provide strategic advice regarding conduct and impact of research in participating communities (18). Informed written consent was obtained from all participants.

## Methods

### Context/setting

In the NT, antenatal care and birthing services are provided by both public and private hospitals in urban settings. Depending on who the local health body is, antenatal care in remote or very remote communities (19) is provided either by Primary Health Care Centres run by NT Health (*n* = 39) or Aboriginal Community Controlled Health Organisations (ACCHOs) (*n* = 133) (20). Importantly, in our setting, place of birth is largely determined by geographical location and risk profile of the women. Publicly funded birthing services are offered predominantly within hospitals, with homebirths only available for a carefully selected number of women within the broader Darwin region (20).

This study consisted of two stages among women living in the Darwin region, or those from a remote community but who were currently in Darwin having relocated for birth. Stage one involved modification and content-validation of the MORi (5) with 15 Australian First Nations women, to facilitate a culturally safe process. Stage two involved piloting and administration of the MORi to 195 women. Participants included Australian First Nations women and women from a migrant or refugee background given that these are the women in our context who experience poorer pregnancy related outcomes compared to other women in Australia and make up a significant proportion of the NT population. The original MORi was developed, validated and used with a diverse cross section of women in Canada, including those from a migrant or refugee background, however without Australian First Nations women (3). Thus, post content-validation in our context with First Nations women, it was determined that our content-validated MORi retained its broad and inclusive design, ensuring it could effectively capture the diverse perspectives of women from culturally and linguistically diverse backgrounds, thus providing confidence that it was appropriate to use with women from a migrant or refugee background without further modification.

### Stage one—modification and content-validation of the MORi

#### Eligibility

First Nations women with a lived experience of pregnancy or birth within the last 12 months, aged ≥18 years, able to converse with research staff in English, who were recruited from an existing qualitative study, with First Nations methodology embedded in the design, being conducted by the authors (21), (*n* = 10) or were receiving antenatal or postnatal care by the First Nations specific Midwifery Group Practice (MGP) at Royal Darwin Hospital (RDH), were eligible to participate. Women who experienced a perinatal death or whose baby had a significant congenital abnormality were excluded.

### Description of MORi

The original MORi (5), created in Canada, through a collaborative process involving healthcare providers, researchers, and women with lived experiences of pregnancy and birth that were culturally secure and relevant. The study was inclusive of diverse communities and reflected the priorities and values of women (5). Thus it was determined to be an appropriate tool to use in our study. The MORi contained 14 items and its responses were recorded on a six-point Likert scale, resulting in a score of 14–84 (6). Scores <32 indicated very low respect, 32–49 low respect, 50–66 moderate respect, and a score of 67–84 indicating high respect.

### Content-validation of the MORi

Content-validity was determined using the content validity index for items (I-CVI) (22), a process where items on the original MORi were individually scrutinized and rated according to their relevance. In this present study, 15 Australian First Nations women individually provided comprehensive feedback about the published items in the MORi (5). The phrasing of items were adapted to suit the context and simplified based on feedback from the women. For example, where the original MORi referenced “midwives and doctors” this was changed to “health staff” to better reflect the reality in the primary health care setting where care of women in pregnancy may involve Aboriginal Health Workers, including Aboriginal Health Practitioners, and Strong Women Workers working in collaboration with Midwives and Doctors (23). Women were individually asked to rate the relevance of each item on the MORi using a 4-point ordinal scale (1= “not relevant”, 2 = “somewhat relevant”, 3 = “quite relevant”, 4 = “highly relevant”). Items with an I-CVI rating of less than 0.78, meaning that at least 2 out of 5 women rated that item as “not relevant” or “somewhat relevant”, were discussed in detail with the women and a member of the First Nations reference group. Alternative phrasing was included in the next iteration. Items with an I-CVI rating of 0.78 or more, meaning that at least 4 of the 5 women in that round rated that item as “quite relevant” or “highly relevant”, were included in the next iteration as they were, or with slightly adapted phrasing to better enable ease of reading. Original items and wording were visible to participants in each subsequent iteration. This process continued until all items had an I-CVI rating of 0.78 or greater.

### Completing the MORi using an avatar

**Figure 1:**
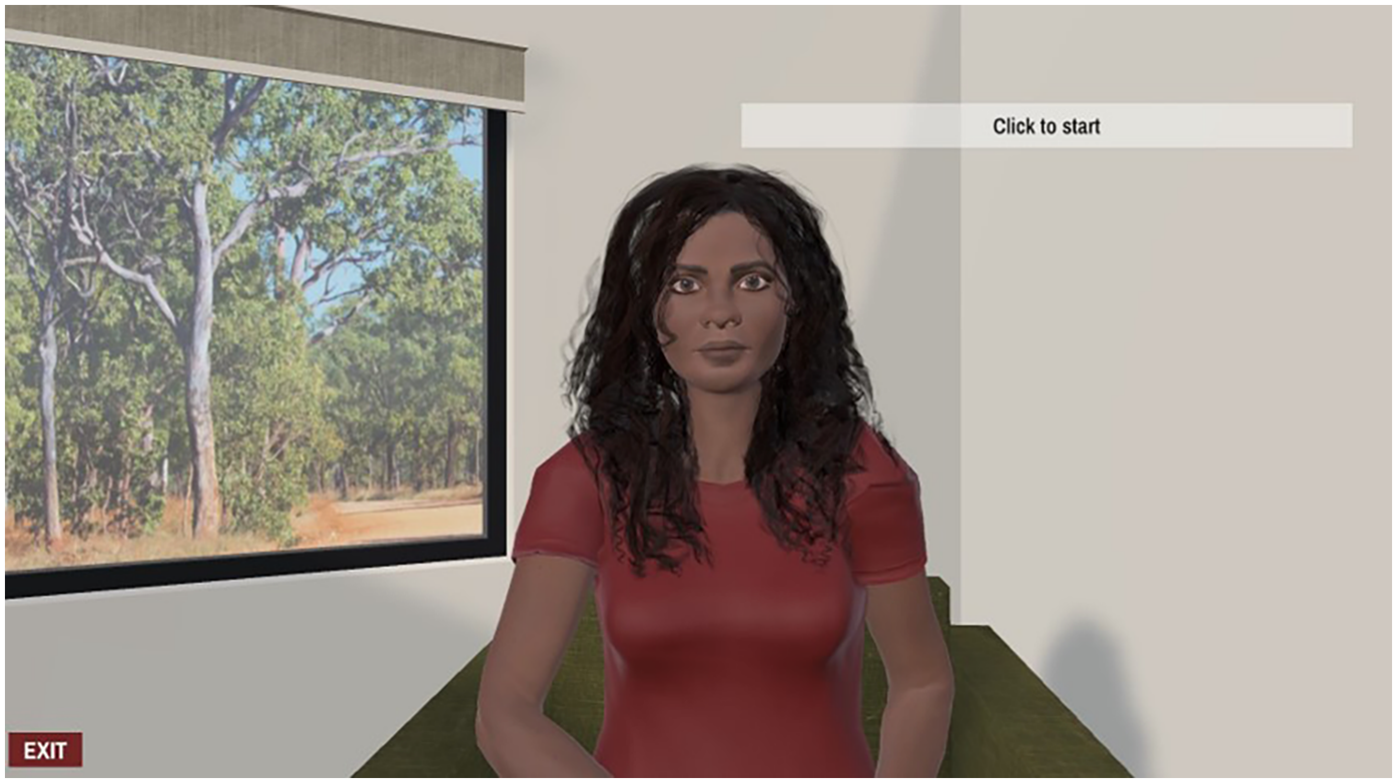
Image of avatar.

Text-based surveys require a substantial level of literacy and comprehension to complete, thus the use of innovative technology to conduct surveys has become increasingly popular in recent years. One such method is the digital avatar, a digital representation of a person or character that is engaging and interactive (23). One of the benefits of using avatars is that they may be designed to facilitate cultural safety, and use multiple languages, which might improve access to people with culturally and linguistically diverse backgrounds. Increasingly, health interventions and research design incorporate the use of avatars to interface with patients or participants, provide education, and collect data. In many cases, use of this technology has been found to be highly feasible, and acceptable to users in a variety of settings (24–26). To maximise participant recruitment, we converted items in the MORi into short stories (see Supplementary Table S1) with the assistance of a privately engaged linguist with decades of experience working with Australian First Nations peoples, and with regular review by members of the First Nations reference group. These stories were then audio recorded in plain English, with the plan to translate and audio record in four First Nations languages. The audio was delivered by the avatar, which we named MORi, via a bespoke software program, also named MORi after the avatar, created using the Unity3D game engine and an authoring tool (27) developed at Macquarie University. The program allowed women who preferred a visual and more engaging way of receiving and responding to the items by interacting with a specially designed digital avatar to participate (Figure 1).

### Stage two—administration of the MORi

#### Eligibility

Australian First Nations women and those from a migrant or refugee background, who spoke either English, Kriol, Murrin Patha, Modern Tiwi, or Anindilyakwa and were within 12 months postpartum, were eligible to participate. These First Nations languages were chosen as they are the main First Nations languages spoken or understood by people in remote regions of the Top End where primary health care is provided by NT Health, as listed on the NT Government “Bushtel” website (28). Exclusion criteria included perinatal death, or the baby having a significant, documented congenital abnormality during this current pregnancy journey.

### Administration of the MORi

Women were recruited using convenience sampling from the postnatal and paediatric wards at RDH, and Community Care Centres in two suburbs within the Darwin region (see Study Flow Chart, Supplementary Figure F1). The maternity and paediatric ward admission lists were reviewed during weekdays for eligible women. Women were only approached after gaining permission from the relevant ward team leaders to ensure women who were unwell, too exhausted, or otherwise inappropriate, were not disturbed. Further, if a woman was engaged in care with a clinician, were sleeping, seemed distracted or very tired, the research team made another time to speak to the woman. In addition, research staff were mindful not to approach women when hospital staff were present. If a staff member entered the room during administration of the MORi, the process was paused until the researcher was alone with the woman to ensure privacy and reduce any potential influence from health care providers. Two child health nurses from the Community Care Centres were trained to screen and recruit women, which included gaining informed consent and administering the text-based version of the MORi.

After obtaining written informed consent, demographic data (e.g., age, education etc.), medical and obstetric history (including current complications), socio-economic status (SEIFA score) and model of care data were collected from women (for full details see Data Collection Form Supplementary Figure F2). The MORi was administered at that time with women either watching the Avatar on a laptop, self-completing the text-based version, or via conversational method with research staff. Women were invited to reflect upon their whole pregnancy journey, including the time of birthing and immediate postpartum period. Designation as having received very low, low, moderate or high respect was determined using the same scale as the original MORi, see Table 1.

**Table 1:** The MORi categories obtained in our study cohort.

MORi category	MORi range	*n* = 195 (%)
Very low respect	14–31	0 (0.0)
Low respect	32–49	3 (1.5)
Moderate respect	50–66	25 (13.0)
High respect	67–84	167 (85.5)

Mothers on respect index (MORi) (3).

The MORi was piloted with first the 50 women by asking for further feedback about the appropriateness and wording of the items.

### Data analysis

We aimed for a total sample size of 160–200 women, based on previous published data (6), but we did not formally calculate a sample size. Previous research used the MORi with a sample size of 161 (6), and successfully demonstrated meaningful differences in respectful maternity care.

Data were entered into a password protected REDCap database (29), accessible only to the research team. Women were assigned a unique study identifier and data were analysed using Stata version 17 (Stata Corp College Station, Texas, USA). Summary statistics are presented as mean and standard deviations (SD), or median and inter-quartile range (IQR 25%–75%) for continuous data dependent on data distribution, and frequency and percentage for categorical data. The Kruskal–Wallis test was used to compare non-normally distributed data across groups.

Univariable and multivariable linear regression analysis were used to assess the relationship between MORi scores and the demographics of the cohort, SEIFA score, antenatal attendance, educational attainment and the primary caregiver.

## Results

### Stage one—modification and content-validation of the MORi

The only major change to the MORi from the original version was Item Ten. The original version said, “*During my pregnancy I felt I was treated poorly by my doctor or midwife because of my type of health insurance or lack of health insurance*.” This item received an I-CVI rating of <0.78 during the first two validation rounds. After further discussion with the women, and seeking advice from members of the First Nations reference group, the phrasing posed to women in the third round of validation was changed to “*During my pregnancy I was not treated well because of where I live/where my community is*” [justified by the use of the socioeconomic index for areas (SEIFA) as a proxy for socioeconomic status] (30). This phrasing received an I-CVI rating of 1.0, consistent with all other items on the MORi, which also received an I-CVI rating of 1.0 on the final iteration. This means that all five women in the third round agreed it was “quite relevant” or “highly relevant”. The final version can be seen below (Table 2). Each iteration can be found in the (Supplementary Tables S2–S4).

**Table 2:** The content-validated MORi.

Item	A: Overall while making decisions during my pregnancy journey: (select or circle one answer for each statement)						
		Strongly disagree	Disagree	Somewhat disagree	Somewhat agree	Agree	Strongly agree
1	I felt comfortable asking questions	1	2	3	4	5	6
2	I felt comfortable saying no to what was suggested	1	2	3	4	5	6
3	I felt comfortable saying yes to what was suggested	1	2	3	4	5	6
4	I felt pushed/humbugged into accepting what was suggested	6	5	4	3	2	1
5	I was able to choose which care options I wanted	1	2	3	4	5	6
6	My personal choices were respected	1	2	3	4	5	6
7	My cultural choices were respected	1	2	3	4	5	6
		Section A total score					
	B: During my pregnancy journey I wasn't treated well: (select or circle one answer for each statement)						
8	Because I am Aboriginal (or because of my nationality)	6	5	4	3	2	1
9	Because of my sexual orientation or gender identity	6	5	4	3	2	1
10	Because of where I live/where my community is	6	5	4	3	2	1
11	Because I didn't agree with the health staff about what to do	6	5	4	3	2	1
		Section B total score					
	C: during my pregnancy journey I didn't ask the questions I wanted to, or didn't talk about things I was worried about: (select or circle one answer for each statement)						
12	Because the health staff were too busy	6	5	4	3	2	1
13	Because I didn't agree with the health staff about what to do	6	5	4	3	2	1
14	Because I was worried that the health staff might think I was being difficult/humbugging them	6	5	4	3	2	1
		Section C total score					

### Stage two—MORi administration

During February to November 2023, 334 women were screened and 195 enrolled (see Table 3—Baseline characteristics). Women self-identified as migrants (*n* = 118), refugees (*n* = 9) or Australian First Nations (*n* = 68). Among First Nations women, 28 were resident of a First Nations community denoted as very remote (19) with the remainder from the broader Darwin region (*n* = 40). Of the women who had migrated to Australia, most were from the Philippines (*n* = 27), India (*n* = 25), and Nepal (*n* = 20), with the remainder from either Southeast Asia, Africa, Europe, or South Pacific countries (*n* = 46). Women who came to Australia seeking refugee status had been born in Africa (*n* = 5), South Asia (*n* = 3) and South America (*n* = 1) (see Supplementary Table S5).

**Table 3:** Baseline characteristics.

Characteristics	*n* = 195 (%)
Demographics	
Age [median (IQR)]	31 (26–34)
Ethnicity	
First Nations	68 (35)
Non-First Nations	127 (65)
Resident of a remote First Nations community	28 (14)
Educational attainment^a^	
Primary school completed	3 (1)
High school completed	66 (34)
Tertiary qualification completed	120 (62)
Current co-morbidities	
None	67 (34)
Autoimmune disease	1 (1)
Thyroid disease	12 (6)
Haematological condition	50 (26)
Heart disease	9 (5)
Renal disease	2 (1)
Liver disease	3 (2)
Respiratory disease	8 (4)
Mental health conditions	23 (12)
Other^b^	64 (33)
Current pregnancy	
Gravidity [median (IQR)]	2 (1–3)
Parity [median (IQR)]	2 (1–2)
Number of living children [mean (SD)]	1.82 (1)
Gestational diabetes	75 (38)
Pregnancy induced hypertension	18 (9)
Infection requiring treatment	44 (23)
History of preterm birth	11 (6)
History of stillbirth	4 (2)
History of caesarean	48 (25)
Number antenatal visits	
None	1 (1)
1–4	10 (5)
5–7	39 (20)
>8	145 (74)
Gestation at first antenatal visit [median (IQR)]	7 (5–12)
Model of care	
Mostly with the same midwife	35 (18)
Mostly with the same small group of midwives	32 (16)
Usually with a different midwife every time	88 (45)
Mostly with the same doctor	29 (15)
Usually with a different doctor every time	53 (27)
Place of antenatal care	
At the primary health centre	41 (21)
At the hospital	133 (68)
At the general practitioner clinic	27 (14)
At home	2 (1)

^a^ Missing data has not been reported as they are minimal and do not significantly impact the overall analysis. IQR, interquartile ratio; SIEFA, socio economic index for areas; SD, standard deviation.

^b^ Other biliary disorders, infections, gynaecological disorders, other endocrine disorders, skin disorders, neurodivergence, hearing or visual impairment, intimate partner violence (see Supplementary Table S6 for detailed list).

Most women (*n* = 184) completed the text-based survey, either by reading and completing it independently or via a conversational method with someone from the research team. Only a small number of women (*n* = 11) elected to watch the avatar, with other women reporting they would find it hard to watch a video while lying down or with frequent interruptions when attending to their babies.

No items were identified as inappropriate, and no new items were posed during the piloting phase.

### MORi score

The median MORi score for all women was 78 (IQR 72–83). Overall, most women reported receiving either “moderate” or “high respect” during their pregnancy journey, with only 3 women reporting “low respect”, and none reporting “very low respect” (Table 1). A graphic representation of these results can be found in the Supplementary Figure F3.

When comparing differences between cohorts (see Table 4), the median MORi score among migrant women was 16.5 points higher compared to First Nations women from remote communities (Interquartile range [IQR], 76–83 vs. 55–76 *p* < 0.001.

**Table 4:** Median mothers on respect index (MORi) scores by cohort.

Cohort	*n* (%)	MORi score median (IQR)	*p* value
First Nations women, remote	28 (14)	63.5 (55–76)	*p* < 0.001
First Nations women, urban	40 (21)	76 (70–83)	
Women from refugee background	118 (60)	79 (76–81)	
Women from migrant background	9 (5)	80 (76–83)	

(MORi), interquartile range (IQR); statistical analysis: Kruskal–Wallis.

Differences in MORi scores were further explored across the SEIFA score, number of antenatal visits attended, educational attainment, and primary caregiver for all cohorts using regression analysis (Table 5). First Nations women from remote communities showed a lower MORi score, while migrant women had a higher MORi score compared to urban First Nations women. A higher MORi score was also associated with a higher SEIFA score and tertiary education (compared to finishing only primary or high school), while the number of antenatal visits and model of care showed no association. Given the overlap in care, model of care groups were combined to mostly the same or mostly different care team (doctors and midwives), or usually the same small group of midwives. Multivariable analysis confirmed lower MORi scores in First Nations remote women compared to urban women, and that higher SEIFA scores were associated with higher MORi scores.

**Table 5:** Association between total MORi score with SEIFA score, antenatal attendances, educational attainment, and primary caregiver.

Univariable analysis	β (95%CI)	*p* value
Cohort		
First Nations women urban	ref	
First Nations women remote	−10.85 (−14.41, −7.30)	<0.001
Women from refugee background	3.19 (0.55, 5.83)	0.018
Women from migrant background	1.13 (−4.19, 6.45)	0.68
Antenatal attendance		
0–4 visits	ref	
5–7 visits	−0.45 (−6.33, 5.44)	0.88
8 or more visits	−1.05 (−6.456, 4.33)	0.70
Educational attainment		
Finished primary or high school	ref	
Finished tertiary education	6.33 (3.88, 8.79)	<0.001
Primary caregiver		
Usually with a different midwife or doctor every time	ref	
Mostly with the same small group of midwives	−3.01 (−6.69, 0.66)	0.11
Mostly with the same midwife or doctor	−1.92 (−4.72, 0.89)	0.18
SEIFA score (per unit)	1.29 (0.95, 1.64)	<0.001
Multivariable analysis	β (95%CI)	*p* value
Cohort		
First Nations women urban	ref	
First Nations women remote	−8.43 (−12.45, −4.14)	<0.001
Women from refugee background	2.42 (−0.25, 5.11)	0.076
Women from migrant background	0.27 (−5.03, 5.57)	0.92
SEIFA score (per unit)	0.53 (0.10, 0.96)	0.016

While the overall median MORi score was high among all women, certain items within the MORi more consistently scored lower (see Table 6). For example, item four, “*Overall while making decisions during my pregnancy journey I felt pushed/humbugged into accepting what was suggested*” had the lowest mean score of 5 (SD 1.5). Women with a refugee background (*n* = 9) had the lowest score [mean 3.78 (SD 2.44)], and migrant women (*n* = 118) had the highest [mean 5.26 (SD 1.28)].

**Table 6:** Mean Likert scale scores for individual MORi items*.*

Item	Individual MORi items	Mean (SD)
	A: overall while making decisions during my pregnancy journey	
1	I felt comfortable asking questions	5.40 (1.06)
2	I felt comfortable saying no to what was suggested	5.06 (1.23)
3	I felt comfortable saying yes to what was suggested	5.41 (0.91)
4	I felt pushed/humbugged into accepting what was suggested	5.00 (1.51)
5	I was able to choose which care options I wanted	5.21 (1.20)
6	my personal choices were respected	5.69 (0.62)
7	my cultural choices were respected	5.62 (0.82)
	B: During my pregnancy journey I wasn't treated well	
8	because I am Aboriginal (or because of my nationality)	5.61 (1.01)
9	because of my sexual orientation or gender identity	5.89 (0.22)
10	because of where I live/where my community is	5.72 (0.60)
11	because I didn't agree with the health staff about what to do	5.60 (0.97)
	C: during my pregnancy I didn't ask the questions I wanted to, or didn't talk about things I was worried about	
12	because the health staff were too busy	5.18 (1.31)
13	because I didn't agree with the health staff about what to do	5.42 (1.05)
14	because I was worried that the health staff might think I was being difficult/humbugging them	5.18 (1.32)

Item two, “*I felt comfortable saying no to what was suggested*” had the second lowest score. For this item, First Nations women who normally reside in a remote community (*n* = 28) scored the lowest [mean 4.43 (SD 1.29)], while migrant women had the highest score [mean 5.24 (SD 1.14)].

Items 12 and 14 each had a mean score of 5.18. First Nations women from remote communities had the lowest score for item 12, “*during my pregnancy I didn't ask the questions I wanted to or didn't talk about things I was worried about because the health staff were too busy*” [mean 3.61 (SD 1.83)], while women from a refugee background had the highest score [mean 5.89 (SD 0.33)]. Similarly, First Nations women from remote communities had the lowest score for item 14, “*during my pregnancy I didn't ask the questions I wanted to, or didn't talk about things I was worried about because I was worried the heath staff might think I was being difficult/humbugging them*” [mean 3.89 (SD 1.87)], and women from a refugee background had the highest score [mean 5.56 (SD 0.73)].

Item five, “*Overall while making decisions during my pregnancy journey I was able to choose which care options I wanted*” had the fifth lowest mean score. First Nations women from remote communities had the lowest score [mean 4.18 (SD 1.47)], with migrant women, interestingly, having the highest score [mean 5.42 (SD 1.03)].

### Limitations

Despite the novelty of our research for the Top End of the NT, our study has several limitations. Firstly, despite extensive efforts, we were unable to translate, and audio record the avatar script into First Nations languages as planned, thus potentially excluding women from some remote and very remote regions of the NT. As only 11 (6%) women chose the avatar to complete the survey, this suggests poor acceptability of this methodology within this cohort of pregnant women, but we do not believe the avatar issue would impact our results. Secondly, we were unable to recruit women from remote and very remote communities serviced by ACCHOs, thus limiting our pool of potential participants. Thirdly, it may be that women who were having a negative experience were those unwilling to participate in this study, thus limiting the generalisability, or gave overly positive responses. Future research may benefit from women being contacted outside the hospital environment, to alleviate any concern about potential bias or fear of negative repercussions. In addition, there were few women from a refugee background represented in the data. This is not surprising considering the relatively small population of Darwin, however, participation of these women may have been higher if recruitment were able to happen via agencies that care for refugee families, although that was not feasible for this study. Also, despite there being extensive First Nations involvement in the design of the study, there were no First Nations researchers involved in data collection, thus limiting the cultural safety of the study. This study assessed content validity using the Content Validity Index for items (I-CVI), ensuring expert consensus on the relevance and clarity of each item. However, while this process strengthens the MORi's validity, it does not establish its full psychometric properties, such as reliability, construct validity, or criterion validity. Further validation studies, including field testing, are needed to confirm the MORi's broader applicability and robustness in different contexts.

## Discussion

We modified and content-validated the MORi to facilitate a culturally safe process for Australian First Nations women in the Top End of the NT using I-CVI (22). To understand the extent of respectful maternity care across the Top End, we administered the MORi to 195 First Nations, migrant or refugee women in their first 12 months postpartum. The overall levels of respectful maternity care reported by these women was relatively high, however, First Nations women from remote communities had a significantly lower median MORi score when compared to other women in the study, aligning with evidence from South Australia which highlights a perceived lack of cultural and psychosocial needs being met (13). This results from this study overall were similar to another study on the East Coast of Australia (6), and a Dutch study from 2020 which also used the MORi (7). Further, responses to individual items are reflective of previous research by the authors exploring pregnant and birthing Australian First Nations women's experiences of care in the Top End of the NT (21, 31) These found that while systematic barriers and government policies negatively impacted their experience of care, women's positive interactions with individual clinicians were at times able to mitigate some of those negative aspects. The high attendance rate for antenatal visits reported in this study suggests that access to care may not be the primary issue in this context. However, the quality of care provided may be of greater significance. This includes the small number of women who reported usually seeing the same midwife.

The WHO defines a positive birthing experience as one that “*fulfills or exceeds a woman's prior personal and socio-cultural beliefs and expectations, including giving birth to a healthy baby in a clinically and psychologically safe environment with continuity of practical and emotional support from a birth companion(s) and kind, technically competent clinical staff*” (1). In this current study, lower scores were more often noted in response to items that highlight the power imbalance found between health care providers and consumers, but which are mitigated in part by high quality continuity models of care. Globally, continuity models of pregnancy and birthing care, primarily, but not exclusively, midwifery led models, have consistently been shown to be highly desirable to women and to result in improved perinatal health outcomes (17, 28, 29, 32). These recurrent interactions between women and the same or small group of carer/s encourages personalized care, facilitates the development of trusting relationships and is empowering for women (27). Continuity models allow care providers to know and more deeply understand women's preferences, provide tailored education, and offer a selection of options suitable for that woman (27). In this environment, women may feel less as though they are being pushed into accepting suggestions that don't align with their preferences, while at the same time giving confidence to care providers that they have provided evidence-based options for women to make an informed choice (27). In addition, having a relationship with the care providers makes time for women to share concerns or worries, and allows them to do so without worrying that they are being a burden. Several studies suggest that women who experience a greater burden of systemic disadvantage, such as Australian First Nations women and women from a refugee background, would benefit greatly from midwifery led continuity models of care in pregnancy (12, 13, 33, 34). The success of this approach hinges on ensuring quality staff, with high levels of cultural security, are available to deliver individualised care which meets the specific needs and preferences of women.

Several models of pregnancy care currently operate out of RDH, including some continuity models such as MGP, and Midwifery in Small Teams. Most women in this study (72%) however, reported usually seeing a different doctor or midwife at each visit, without the option of a continuity model. Importantly, some women from a migrant background in our setting have limited options for pregnancy care as visa restrictions often do not include access to Medicare (universal healthcare afforded to Australian citizens). This limited some women's options for any model of pregnancy care within the public system due to out-of-pocket expenses incurred at a public facility. Literature from other high-income countries shows that access to women's health services, including pre-conception care and family planning, are restricted when women don't have access to services that provide free health care, such as Medicare, thus increasing the risk of adverse outcomes (35, 36).

Importantly, those who scored lowest on the MORi, and on four of the five lowest scoring items were First Nations women who usually resided in a remote community, which have lower SEIFA scores. Studies from contexts as diverse as Singapore (37), and California (38) found that socioeconomic vulnerability was associated with poorer pregnancy outcomes. However, these disparities were not driven solely by income. In Singapore (37); fear of stigma about poverty, and guilt about being perceived as a “bad mother,” led women to seek advice from family and friends or social media rather than health professionals. In California (38), a lack of agency to make own decisions about mode of birth and care provider impacted women's experience of care. Similarly, our study reflects these findings, highlighting how socioeconomic circumstances affect a woman's ability to build respectful relationships with care providers. In our context, women from remote communities are restricted to primary care services provided by the local health clinic which do not include birthing services and sometimes by “fly-in-fly-out” models of pregnancy care where care is provided intermittently by visiting clinicians. Furthermore, these women are requested to leave their community at around 37 weeks gestation and relocate to a tertiary centre for the birth. While many of these women are accepted into the MGP team operating out of RDH, their overall care can only be described as fragmented. New relationships are having to be developed in a short period of time, which may inhibit the development of trust, and care providers' ability to fully appreciate these women's preferences. In addition, women from these communities with risk factors are often expected to relocate even earlier during the pregnancy to access specialist services, sometimes unaccompanied by a family member for many weeks at a time, further increasing their risk of a negative experience.

The presence of a companion during pregnancy and birth is of vital importance. The WHO (1), and others (39, 40) recommend women have a companion of choice with them throughout labour and birthing, as it improves a woman's experience of care, improves clinical outcomes for women and babies and even reduces a woman's perception of pain, thus improving her experience. In our setting, women who are required to relocate for birth are only eligible to have a companion accompany them if they are experiencing a “complex” pregnancy, or have another child under 2 years of age who will accompany them (41). This policy leaves a significant number of women ineligible and thus unable to have a companion of choice with them during the later stages of their pregnancy or while they are birthing. In addition to the benefits already outlined, companions in pregnancy and birth can also act as an advocate for women. Women who felt pushed into accepting what was suggested by health care providers, felt uncomfortable saying no to what was suggested, were reluctant to ask questions or talk about their worries because staff were too busy or the woman was worried about being perceived as being difficult, would greatly benefit from having a companion who understands their preferences, and is able to advocate for her at a vulnerable time. Universal access to continuity of carer models, companions for all women, and reliable access to interpreters would address some of the factors that impact a woman's experience of respectful maternity care.

## Conclusions

Among the women participating in this study, the level of respectful maternity care experienced was relatively high. Among the cohorts, Australian First Nations women from very remote communities reported experiencing the lowest levels of respectful maternity care, which may be the result of ongoing systemic racism faced by these women. Further, special attention is required for women from a refugee background to ensure that they feel free to make fully informed choices. More work is needed to ensure that not only individual clinicians, but crucially the systems designed to care for pregnant and birthing women are provided in a manner “*to all women that maintains their dignity, privacy, and confidentiality, ensures freedom from harm and mistreatment, and enables informed choice and continuous support during labour and childbirth*” (1).

## Data Availability

The raw data supporting the conclusions of this article will be made available by the authors, without undue reservation.
