# Supplementary material for: Content validation and use of mothers on respect index to determine levels of respectful maternity care among women facing disadvantage, birthing in the Top End of the Northern Territory: a cross-sectional study
Source: Front Glob Womens Health. 2025 Apr 3;6:1531904. doi: 10.3389/fgwh.2025.1531904 (PMC12003359; doi:10.3389/fgwh.2025.1531904)
Supplement: Supplementary file 1 [file Table1.docx]

About the authors

The lead author, ERB, is a non-First Nations nurse and midwife who has lived and worked in the Northern Territory for over eight years, four and a half of which were spent on Yolngu Country (Nhulunbuy), and four on Larrakia Country (Darwin), where she worked in midwifery and research alongside both First Nations and non-First Nations women. MRT, an experienced and well-respected qualitative researcher living in Sydney, is a Kooma/Euahlayi woman. ABC, a non-First Nations medical doctor and researcher, has over 25 years of experience providing clinical services to First Nations children in rural and remote regions of the NT, Central Australia, and Queensland. GBM, also a non-First Nations nurse and researcher, has decades of experience working with First Nations children and their families in urban, rural, and remote regions of the NT and Central Australia. RLW, a non-First Nations woman and researcher, has lived and worked in the Northern Territory for many decades and has established significant personal and professional relationships with numerous Australian First Nations communities. MP is an experienced senior research programmer with over 10 years of experience working at Macquarie University on Virtual Reality, Game Engine technology, and Web-based projects who implemented the avatar. DR is a professor in the School of Computing at Macquarie University whose multidisciplinary research focuses on the use of avatars, better described as embodied conversational agents, for a wide range of health and psychoeducation contexts with the aim of empowering users and encouraging positive behaviour change.

Figure F1, Study Flow Chart

no

Thank woman, wish them all the best, and leave

Initial screening

- Handover sheet from postnatal and paediatric wards
- Child Health Nurse clinic list

Secondary screening

- Check medical notes for suburb and nationality only
- Check appropriateness with midwife or nurse caring for woman

Determine appropriateness of immediate approach

- With clinician or visitor
- Sleeping
- Visually busy or distressed

Invite woman to participate

Delay approach/ return later

Decline

Accept

- Introduce self
- Explain purpose of study
- Negotiate best time to complete

Proceed immediately

- Gain informed consent
- Gather demographic data
- Administer MORi
- Review medical notes

Congratulate woman on birth, wish them all the best, and leave

Return later

Figure F2, Data Collection Tool


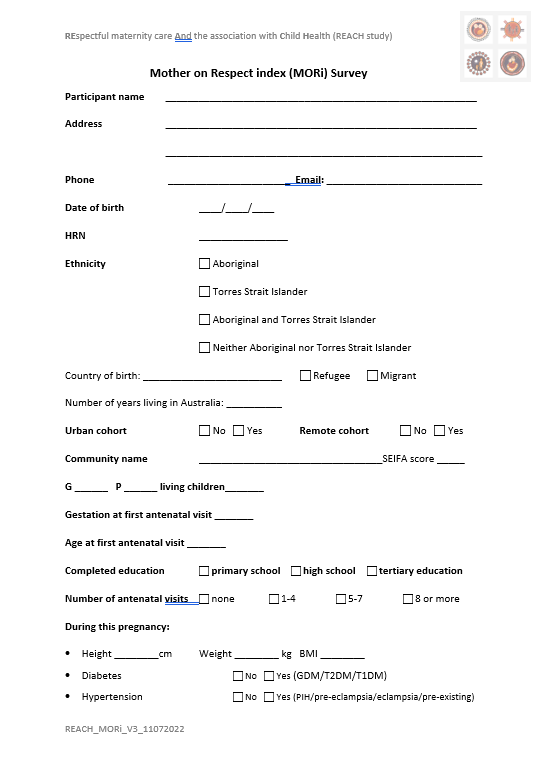

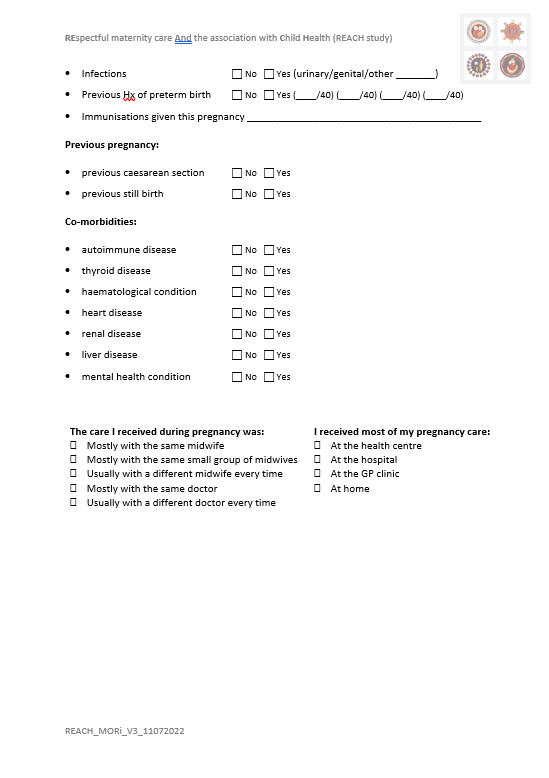


Table S1 MORi survey script

| On this screen we have a story for you to hear. We invite you to listen to the story then answer a question about what happened to you when you were carrying a baby inside you and when you were having those baby pains. The people who looked after you when you were carrying a baby inside you and when you were having those baby pains will not know that you answered these questions. You can answer yes or no, or you can ask to hear the story again. There is no right or wrong answer. You choose whichever answer is best for you. Your story is safe. | Introduction |
| --- | --- |
| “When I was was carrying this baby inside me and when I was having those baby pains, I had some problems and had to decide what to do. Sometimes my problem was like swelling in the legs or morning sickness, and there were other problems too. I didn’t know what to do so I asked the midwife. When you were carrying that baby inside you or when you were having those baby pains did you feel happy about asking the doctor or midwife what to do?” | **Overall while making decisions during my pregnancy journey:**  I felt comfortable asking questions |
| “Sometimes when I asked these questions, I did not like the answer the midwife gave me, so I said no. The midwife didn’t make me feel bad for saying no. When you were carrying that baby inside you or when you were having those baby pains did you feel happy to say no to what the doctor or midwife asked of you?” | **Overall while making decisions during my pregnancy journey:**  I felt comfortable saying no to what was suggested |
| “Other times when I asked those questions, I did like the answer the midwife gave me. I thought they were good ideas, so I said yes. When you were carrying that baby inside you or when you were having those baby pains did you feel happy to say yes to what the doctor or the midwife asked of you?” | **Overall while making decisions during my pregnancy journey:**  I felt comfortable saying yes to what was suggested |
| “When I was when I was carrying this baby inside me and when I was having those baby pains, there were times that the midwife was humbugging me to do something for myself or my baby that I did not like or did not understand. When you were carrying that baby inside you or when you were having those baby pains did you get humbugged by the doctor or midwife to do something you did not want to do or to do something you did not understand?” | **Overall while making decisions during my pregnancy journey:**  I felt pushed/humbugged into accepting what was suggested |
| “When I found out I was having this baby, I knew the best thing for me was to have check ups with the midwife called Katie. There was another midwife at the clinic too, but I didn’t want to see her. The clinic said that was ok, and I had all my check-ups with Katie. I was real happy about that. When you were carrying that baby inside you or when you were having those baby pains could you pick the person that you wanted to do your check-ups or the person that you wanted to look after you?” | **Overall while making decisions during my pregnancy journey:**  I was able to choose which care option I wanted |
| “When I was having those baby pains, I didn’t want to have that medicine in the back called an epidural. I was sure about that. When the baby was coming out my pain was really bad. The midwife asked me if I wanted the epidural, but I said no. She did not humbug me to take the medicine, she respected what I said. When you were carrying that baby inside you or when you were having those baby pains did the doctors and the midwives respect what you said?” | **Overall while making decisions during my pregnancy journey:**  My personal choices were respected |
| “When I was carrying this baby inside of me, a member of my family passed away. It was really important for me to be a part of the sorry business. It was a long way from the clinic and it meant I was going to be away for a while. The midwife helped me to be safe and changed my checkups for me. I was happy that she understood what I needed to do. When you were carrying that baby inside you or when you were having those baby pains did the doctors and the midwives respect your culture?” | **Overall while making decisions during my pregnancy journey:**  My cultural choices were respected |
| “Sometimes, I was treated badly by the midwives and doctors because I’m Aboriginal. I know that’s happened to other mob too. When you were carrying that baby inside you or when you were having those baby pains did you ever get treated badly by the doctor or midwife because you’re Aboriginal?” | **During my pregnancy journey I wasn’t treated well:**  Because I am Aboriginal |
| “I know two women who love each other and got married. They used IVF and had a baby. They were so happy, but sometimes they were treated badly by the midwives and doctors because they were two ladies having a baby together instead of a lady with a man. When you were carrying that baby inside you or when you were having those baby pains did you ever get treated badly because you were having a baby with another lady instead of with a man?” | **During my pregnancy journey I wasn’t treated well:**  Because of my sexual orientation or gender identity |
| “Other times I got treated badly by the midwives and doctors because of the place where my community is. Maybe they thought it was a bad place to live. They made me feel shame. When you were carrying that baby inside you or when you were having those baby pains did you ever get treated badly or made to feel shame because of the place where your community is?” | **During my pregnancy journey I wasn’t treated well:**  Because of where I live/where my community is |
| “When I was in the hospital, I knew what my baby needed to be safe and healthy but the doctors said that wasn’t right. They made me feel bad, but I knew I was right. When you were carrying that baby inside you or when you were having those baby pains did the doctor or midwife ever make you feel like something was bad or a shame job even though you knew you were right?” | **During my pregnancy journey I wasn’t treated well:**  Because I didn’t agree with the health staff about what to do |
| “My sister had a baby, she said the midwives were so busy, rushing around. She was worried about something, but they were busy and she didn’t want to humbug them. When you were carrying that baby inside you or when you were having those baby pains were you ever worried about something but couldn’t ask the midwives or doctors because they were too busy?” | **During my pregnancy journey I didn’t ask the questions I wanted to, or didn’t talk about things I was worried about:**  Because the health staff were too busy |
| “My other sister was carrying a baby inside her. She knew what she needed to do to be safe and healthy, but the doctors at the hospital said she was wrong. So she didn’t talk with them about what she was worried about, she just kept all the worry inside herself. When you were carrying that baby inside you or when you were having those baby pains was there a time time when you did not ask the midwife or doctor a question or you did not talk to them about what you were worried about because you thought they would say you were wrong?” | **During my pregnancy journey I didn’t ask the questions I wanted to, or didn’t talk about things I was worried about:**  Because I didn’t agree with the health staff about what to do |
| “After my baby came out, I had lots of pain and needed some medicine. I was too scared to ask the midwife about it because I thought they might not like me humbugging them, so I didn’t ask. When you were carrying that baby inside you or when you were having those baby pains, did you ever worry that you were humbugging the midwives or doctors?” | **During my pregnancy journey I didn’t ask the questions I wanted to, or didn’t talk about things I was worried about:**  Because I was worried the health staff might think I was being difficult/humbugging them |
| Thank you so much for sharing part of your story. I will keep it safe. | Finishing screen |

Table S2: Original MORi

| **A: overall while making decisions about my pregnancy or birth care:** | | | | |
| --- | --- | --- | --- | --- |
|  | Not relevant | Somewhat relevant | Quite relevant | Highly relevant |
| I felt comfortable asking questions | 1 | 2 | 3 | 4 |
|  | | | | |
| I felt comfortable declining care that was offered | 1 | 2 | 3 | 4 |
|  | | | | |
| I felt comfortable accepting the options for care that my doctor or midwife recommended | 1 | 2 | 3 | 4 |
|  | | | | |
| I felt pushed into accepting the options my midwife or doctor suggested | 1 | 2 | 3 | 4 |
|  | | | | |
| I chose the care options that I received | 1 | 2 | 3 | 4 |
|  | | | | |
| My personal preferences were respected | 1 | 2 | 3 | 4 |
|  | | | | |
| My cultural preferences were respected | 1 | 2 | 3 | 4 |
|  | | | | |
| **B: During my pregnancy I felt that I was treated poorly by my doctor or midwife because of:** | | | | |
|  | Not relevant | Somewhat relevant | Quite relevant | Highly relevant |
| My race, ethnicity, cultural background or language | 1 | 2 | 3 | 4 |
|  | | | | |
| My sexual orientation and/ or gender identity | 1 | 2 | 3 | 4 |
|  | | | | |
| My type of health insurance or lack of insurance | 1 | 2 | 3 | 4 |
|  | | | | |
| A difference of opinion with my caregivers about the right care for myself or my baby | 1 | 2 | 3 | 4 |
|  | | | | |
| **C: during my pregnancy I held back from asking questions or discussing my concerns because:** | | | | |
|  | Not relevant | Somewhat relevant | Quite relevant | Highly relevant |
| My doctor or midwife seemed rushed | 1 | 2 | 3 | 4 |
|  | | | | |
| I wanted maternity care that differed from what my doctor or midwife recommended | 1 | 2 | 3 | 4 |
|  | | | | |
| I thought my doctor or midwife might think I was being difficult | 1 | 2 | 3 | 4 |
|  | | | | |

Table S3 MORi first iteration

| **A: overall while making decisions about my pregnancy or birth care:** | Not relevant | Somewhat relevant | Quite relevant | Highly relevant |
| --- | --- | --- | --- | --- |
| I felt comfortable asking questions | 1 | 2 | 3 | 4 |
|  | | | | |
| I felt comfortable saying no to care that was offered | 1 | 2 | 3 | 4 |
| *~~I felt comfortable declining care that was offered~~* | | | | |
| I felt comfortable to accept the care that was recommended. ~~accepting the options for care that my doctor or midwife recommended~~ | 1 | 2 | 3 | 4 |
|  | | | | |
| I felt pushed into accepting what was suggested. ~~options my midwife or doctor suggested~~ | 1 | 2 | 3 | 4 |
|  | | | | |
| I received the care options I chose | 1 | 2 | 3 | 4 |
| *~~I chose the care options that I received~~* | | | | |
| My personal preferences were respected | 1 | 2 | 3 | 4 |
|  | | | | |
| My cultural preferences were respected | 1 | 2 | 3 | 4 |
|  | | | | |
| **B: During my pregnancy I felt that I wasn’t treated well because of: ~~treated poorly by my doctor or midwife because of:~~** | Not relevant | Somewhat relevant | Quite relevant | Highly relevant |
| My race, ethnicity, cultural background or language | 1 | 2 | 3 | 4 |
|  | | | | |
| My sexual orientation and/ or gender identity | 1 | 2 | 3 | 4 |
|  | | | | |
| My socio-economic situation | 1 | 2 | 3 | 4 |
| *~~My type of health insurance or lack of insurance~~* | | | | |
| We had different ideas about what was the right thing to do for me or my baby ~~A difference of opinion with my caregivers about the right care for myself or my baby~~ | 1 | 2 | 3 | 4 |
|  | | | | |
| **C: during my pregnancy I didn’t ask the questions I wanted to or didn’t discuss things I was concerned about because: ~~held back from asking questions or discussing my concerns because:~~** | Not relevant | Somewhat relevant | Quite relevant | Highly relevant |
| My doctor or midwife seemed rushed | 1 | 2 | 3 | 4 |
|  | | | | |
| I wanted a different type of care to what they recommended. ~~maternity care that differed from what my doctor or midwife recommended~~ | 1 | 2 | 3 | 4 |
|  | | | | |
| I thought my doctor or midwife might think I was being difficult | 1 | 2 | 3 | 4 |
|  | | | | |

Table S4 MORi second iteration

| **A: overall while making decisions during my pregnancy journey:** | Not relevant | Somewhat relevant | Quite relevant | Highly relevant |
| --- | --- | --- | --- | --- |
| I felt comfortable asking questions | 1 | 2 | 3 | 4 |
|  | | | | |
| I felt comfortable saying no to what was suggested | 1 | 2 | 3 | 4 |
| ~~I felt comfortable saying no to care that was offered~~ | | | | |
| I felt comfortable saying yes to what was suggested | 1 | 2 | 3 | 4 |
| ~~I felt comfortable to accept the care that was recommended~~. | | | | |
| I felt pushed/humbugged into accepting what was suggested | 1 | 2 | 3 | 4 |
| ~~I felt pushed into accepting what was suggested.~~ | | | | |
| I was able to choose which care option I wanted | 1 | 2 | 3 | 4 |
| ~~I received the care options I chose~~ | | | | |
| My personal choices were respected | 1 | 2 | 3 | 4 |
|  | | | | |
| My cultural choices were respected | 1 | 2 | 3 | 4 |
|  | | | | |
| **B: During my pregnancy journey I wasn’t treated well:** | Not relevant | Somewhat relevant | Quite relevant | Highly relevant |
| Because I am Aboriginal and/or Torres Strait Islander (or because of my nationality) | 1 | 2 | 3 | 4 |
| ~~My race, ethnicity, cultural background or language~~ | | | | |
| Because of my sexual orientation or gender identity | 1 | 2 | 3 | 4 |
|  | | | | |
| Because of where I live/where my community is | 1 | 2 | 3 | 4 |
| ~~My socio-economic situation~~ | | | | |
| Because I didn’t agree with the health staff about what to do | 1 | 2 | 3 | 4 |
| ~~We had different ideas about what was the right thing to do for me or my baby~~ | | | | |
| **C: during my pregnancy journey I didn’t ask the questions I wanted to, or didn’t talk about things I was worried about:** | Not relevant | Somewhat relevant | Quite relevant | Highly relevant |
| Because the health staff were too busy | 1 | 2 | 3 | 4 |
| ~~My doctor or midwife seemed rushed~~ | | | | |
| Because I didn’t agree with the health staff about what to do | 1 | 2 | 3 | 4 |
| ~~I wanted a different type of care to what they recommended.~~ | | | | |
| Because I was worried the health staff might think I was being difficult/humbugging them | 1 | 2 | 3 | 4 |
| ~~I thought my doctor or midwife might think I was being difficult~~ | | | | |

Figure F3, MORi Scores Graph


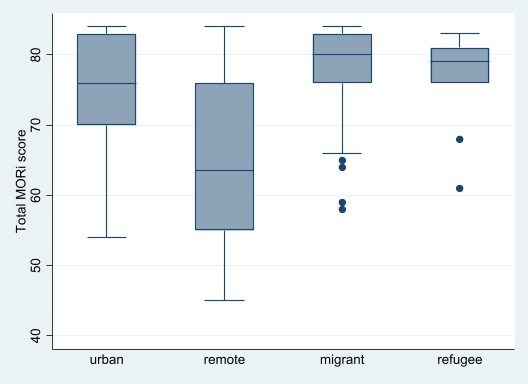


Table S5 Country of birth, migrant and refugee women

| **Country of birth** | **Frequency (%)** |
| --- | --- |
| Bangladesh | 3 |
| Bulgaria | 1 |
| Cambodia | 2 |
| China | 2 |
| Columbia | 2 |
| Egypt | 1 |
| Ghana | 2 |
| Greece | 1 |
| India | 25 |
| Indonesia | 4 |
| Italy | 1 |
| Kenya | 4 |
| Malaysia | 1 |
| Nepal | 20 |
| New Zealand (Mouri) | 1 |
| Nigeria | 3 |
| Pakistan | 4 |
| Peru | 1 |
| Philippines | 27 |
| South Sudan | 1 |
| Sri Lanka | 7 |
| Sudan | 1 |
| Thailand | 1 |
| The Congo | 3 |
| Timor-Leste | 1 |
| United Arab Emirates | 1 |
| Vietnam | 5 |
| Zimbabwe | 2 |

Table S6 comorbidities - other

| Co-morbidities | Frequency |
| --- | --- |
| Antepartum haemorrhage | 3 |
| Attention deficit hyperactivity disorder | 1 |
| Cholestasis | 3 |
| Chorioamnionitis | 2 |
| Dermatitis | 1 |
| Domestic violence from current partner | 2 |
| Eczema | 1 |
| Endometriosis | 1 |
| Fibroids | 2 |
| Genital tract infection | 6 |
| Hearing impairment | 3 |
| Holt-Oram syndrome | 1 |
| Hx Tuberculosis | 4 |
| Idiopathic intracranial hypertension | 1 |
| Illicit drug use | 3 |
| Intra uterine growth restriction | 1 |
| Irritable bowel syndrome | 1 |
| In vitro fertilisation (IVF) pregnancy | 2 |
| Migraines | 3 |
| Oligohydramnios | 2 |
| Pancreatitis | 1 |
| Placenta accreta | 1 |
| Placental insufficiency | 1 |
| Poly cystic ovary syndrome | 6 |
| Pulmonary hypertension | 1 |
| Raynaud's disease | 1 |
| Shortened cervix | 6 |
| Scoliosis | 2 |
| Social pressures | 1 |
| Two vessel Cord | 1 |
| Visual impairment | 1 |
